# Supplementary material for: Anserine and Carnosine Induce HSP70-Dependent H2S Formation in Endothelial Cells and Murine Kidney
Source: Antioxidants (Basel). 2022 Dec 28;12(1):66. doi: 10.3390/antiox12010066 (PMC9855136; doi:10.3390/antiox12010066)
Supplement: Supplementary file 1 [file antioxidants-12-00066-s001.zip › antioxidants-2028948-supplementary.pdf]

Supplementary Tables and Figures

Supplementary Tables

Supplementary Table S1: H_2S formation from homocysteine increases H_2S formation dose-dependently in HK-2 cells (n=3)

Homocysteine	H_2S formation [pmol/mg]
0.1 mM	6.0 ± 1.0
1 mM	8.5 ± 0.3 **
2.5 mM	12.0 ± 0.1 ****
5 mM	14.5 ± 0.8 ****

**=p < 0.01; ****=p < 0.0001 compared to control (one-way ANOVA with Tukey's test).

Supplementary Table S2: H_2S formation from homocysteine (5 mM) in HK-2 cells and different endothelial cell types (n=3-6)

	H_2S formation [pmol/mg]			
Cell type	Control	+ Anserine (1 mM)	+ Carnosine (1 mM)	+ Anserine (1 mM) + Carnosine (1 mM)
HK-2	15.0 ± 0.8	22.9 ± 1.4 ****	18.9 ± 1.1 **	22.6 ± 0.5 ****
HUVEC	15.4 ± 0.1	23.9 ± 2.3 ***	13.9 ± 1.5	23.0 ± 2.0 **
HUAEC	21.1 ± 1.4	27.6 ± 0.5 ****	19.5 ± 0.4	26.5 ± 0.4 ***
MCEC	72.3 ± 9.4	111.3 ± 15.8 ***	64.7 ± 5.3	118.9 ± 15.5 ***

**=p < 0.01; ***=p < 0.001; ****=p < 0.0001 compared to respective control (one-way ANOVA with Tukey's test).

Supplementary Table S3: Anserine increases H_2S formation from homocysteine (5 mM) dose-dependently in HK-2 cells (n=3)

		H_2S formation [pmol/mg]
Control		14.8 ± 0.8
+ Anserine	0.5 mM	16.2 ± 0.7
	1 mM	23.7 ± 0.5 ****
	2.5 mM	29.1 ± 1.6 ****
+ Carnosine	0.5 mM	14.4 ± 0.4
	1 mM	19.6 ± 1.2 ***
	2.5 mM	21.4 ± 1.0 ****
+ Anserine + Carnosine	0.5 mM	16.0 ± 0.3
	1 mM	23.8 ± 0.7 ****
	2.5 mM	28.6 ± 1.4 ****

***=p < 0.001; ****=p < 0.0001 compared to control (one-way ANOVA with Tukey's test).

Supplementary Table S4: Addition of Na_2S to serum inhibits CN1 activity dose-dependently (n=3-8)

	Control	0.1 mM Na_2S	0.2 mM Na_2S	0.5 mM Na_2S	1 mM Na_2S
CN1 carnosine degradation activity [µmol/ml × h^{-1}]	2.00 ± 0.26	0.53 ± 0.23 ****	0.24 ± 0.09 ****	0.07 ± 0.02 ****	0.05 ± 0.02 ****
CN1 anserine degradation activity [µmol/ml × h^{-1}]	2.03 ± 0.26	0.49 ± 0.21 ****	0.23 ± 0.09 ****	0.05 ± 0.03 ****	0.05 ± 0.02 ****

****=p < 0.0001 compared to control (one-way ANOVA with Tukey's test).

Supplementary Table S5: Rescue of the *Hsp70*-KO by *Hsp70*-transfection (clone 1-3) restored H_2S formation to the level of WT MCEC (n=3-9)

	H_2S formation [pmol/mg]			
	Control	+ Anserine (1 mM)	+ Carnosine (1 mM)	+ Anserine (1 mM) + Carnosine (1 mM)
MCEC WT	72.3 ± 9.4 ****	111.3 ± 15.8 ****	64.7 ± 5.3 **	118.9 ± 15.5 ****
MCEC *Hsp70*-KO	42.8 ± 6.4	47.0 ± 8.9	44.3 ± 7.0	35.7 ± 5.3
Clone 1	72.0 ± 5.4 ***	106.0 ± 6.8 ****	65.5 ± 2.6 **	110.4 ± 9.5 ****
Clone 2	68.2 ± 7.9 ***	88.9 ± 5.5 **	66.8 ± 5.4 **	87.0 ± 4.5 ***
Clone 3	71.4 ± 9.8 ***	99.5 ± 5.6 ***	67.9 ± 5.4 ***	93.9 ± 3.2 ****

**=p < 0.01; ***=p < 0.001; ****=p < 0.0001 compared to respective MCEC *Hsp70*-KO treatment (one-way ANOVA with Tukey's test).

Supplementary Table S6: Renal H_2S formation in *Cndp1*-KO and WT mice from homocysteine (47- to 51-week-old mice, n=3) and form cysteine under fasting and non-fasting conditions (23- to 25-week-old mice, n=3-5).

	H_2S formation [pmol/mg]			
	+ Homocysteine (5mM)	+ Homocysteine (10mM)	Non-fasting (2.5 mM cysteine)	Fasting (2.5 mM cysteine)
WT f	1.8 ± 0.9	3.4 ± 0.5	10.6 ± 1.4	10.8 ± 1.3
WT m	0.8 ± 0.04	1.3 ± 0.3	11.1 ± 1.1	8.4 ± 1.2
Cndp1-KO f	5.6 ± 1.6 **	8.8 ± 1.5 ***	20.7 ± 5.7 *	22.0 ± 3.4 ****
Cndp1-KO m	1.9 ± 0.5	2.2 ± 0.8	9.7 ± 2.2	9.6 ± 1.7

*=p < 0.05; **=p < 0.01; ***=p < 0.001; ****=p < 0.0001 compared to control (one-way ANOVA with Tukey's test).

Supplementary Table S7: H_2S formation was reduced by CSE and CBS inhibitors in kidney tissue lysate of WT and *Cndp1*-KO mice (47- to 51-week-old, n=3-5)

	H_2S formation [pmol/mg]			
	Control	+ PAG (0.315 mM)	+ AOAA (0.0315 mM)	+ PAG (0.315 mM)<br>+ AOAA (0.0315 mM)
WT f	11.55 ± 2.01	6.73 ± 1.74 **	0.23 ± 0.45 ****	0.77 ± 1.40 ****
WT m	11.97 ± 0.78	9.24 ± 2.38 *	0.78 ± 0.16 ****	0.99 ± 0.30 ****
Cndp1-KO f	24.42 ± 6.43	15.07 ± 2.25	3.27 ± 1.37 ***	1.30 ± 2.25 ****
Cndp1-KO m	7.22 ± 0.70	5.51 ± 1.49	0.80 ± 0.40 ****	0.59 ± 0.35 ****

*=p < 0.05; **=p < 0.01; ***=p < 0.001; ****=p < 0.0001 compared to respective control (one-way ANOVA with Tukey's test).

Supplementary Table S8: Renal H_2S formation and anserine concentrations in *Hsp70*-KO mice (23- to 28-week-old, n=6-9)

	H_2S formation [pmol/mg protein]	Anserine [nnmol/mg protein]
WT f	12.2 ± 1.6	0.3 ± 0.2
WT m	8.5 ± 1.5	0.2 ± 0.1
*Hsp*70-KO f	6.1 ± 0.7 ****	0.3 ± 0.2
*Hsp*70-KO m	7.3 ± 0.8	0.4 ± 0.2

****=p < 0.0001 compared to respective WT (one-way ANOVA with Tukey's test).

Supplementary Table S9: Incubation of kidney tissue lysate with anserine and carnosine (6h) did not increase renal H_2S formation in WT mice (23- to 26-week-old, n=3-5)

	H_2S formation [pmol/mg]	
	+Anserine (2.5mM)	+Carnosine (2.5mM)
WT f	10.1 ± 0.5	9.9 ± 0.7
WT m	9.7 ± 0.6	9.2 ± 0.1

Supplementary Figures

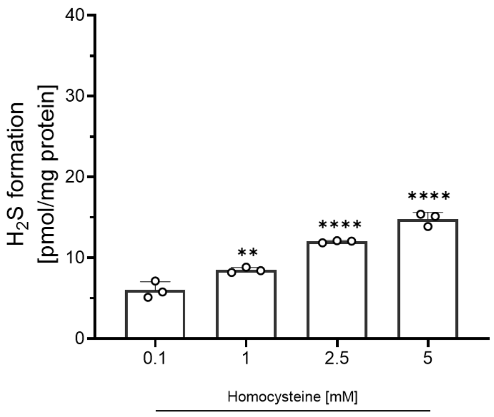

Supplementary Figure S1: Homocysteine dependent H_2S formation in human proximal tubular epithelial cells (HK-2)

Homocysteine dose-dependently increased H_2S formation in HK-2 cells compared to untreated cells. **: p<0.01; ****: p<0.0001 (one-way ANOVA with Tukey's test).

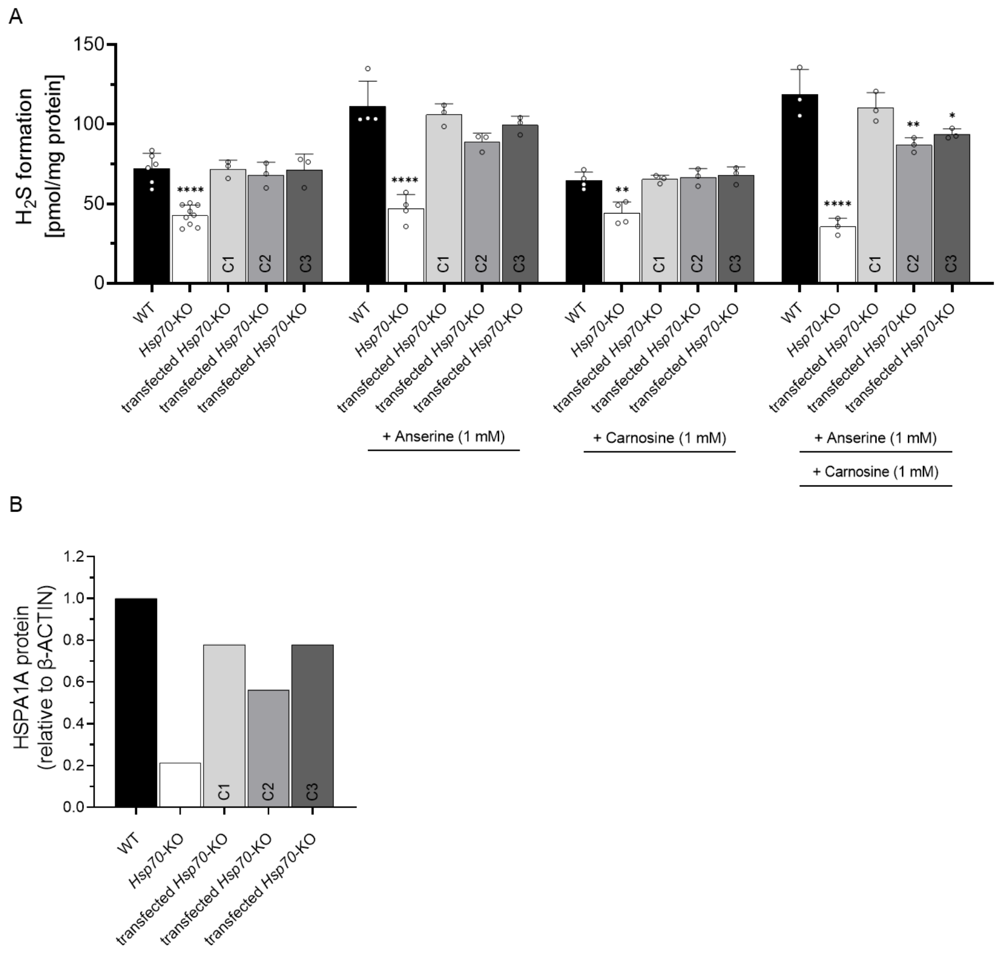

Supplementary Figure S2: H_2S formation depends on HSP70

A. Transfection of *Hsp70* restored H_2S formation in all three *Hsp70*-KO clones.

B. Successful transfection of *Hsp70* was reconfirmed in all three clones by western blot. C1-3: clone 1-3.

*: $p<0.05$;   **: $p<0.01$(one-way ANOVA with Tukey's test)

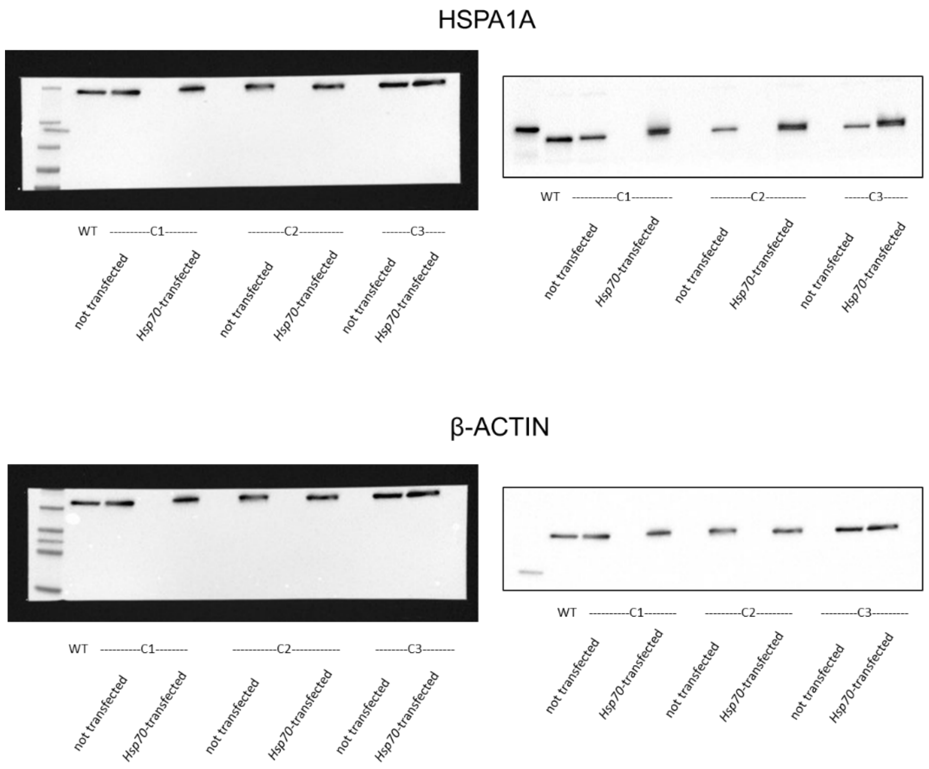

Supplementary Figure S3: Original western blot membranes of protein expression of HSPA1A and β-ACTIN in cell clones

Sample order from left to right:

WT, clone 1 (C1), *Hsp70*-transfected clone 1, clone 2 (C2), *Hsp70*-transfected clone 2, clone 3 (C3), *Hsp70*-transfected clone 3

left panel: membrane merged with marker (iBright Prestained Protein Ladder; Thermo Fisher Scientific)

right panel: 30 kDa and 80 kDa immunodetectable bands contain IgG binding sites that can be visualized simultaneously with target protein

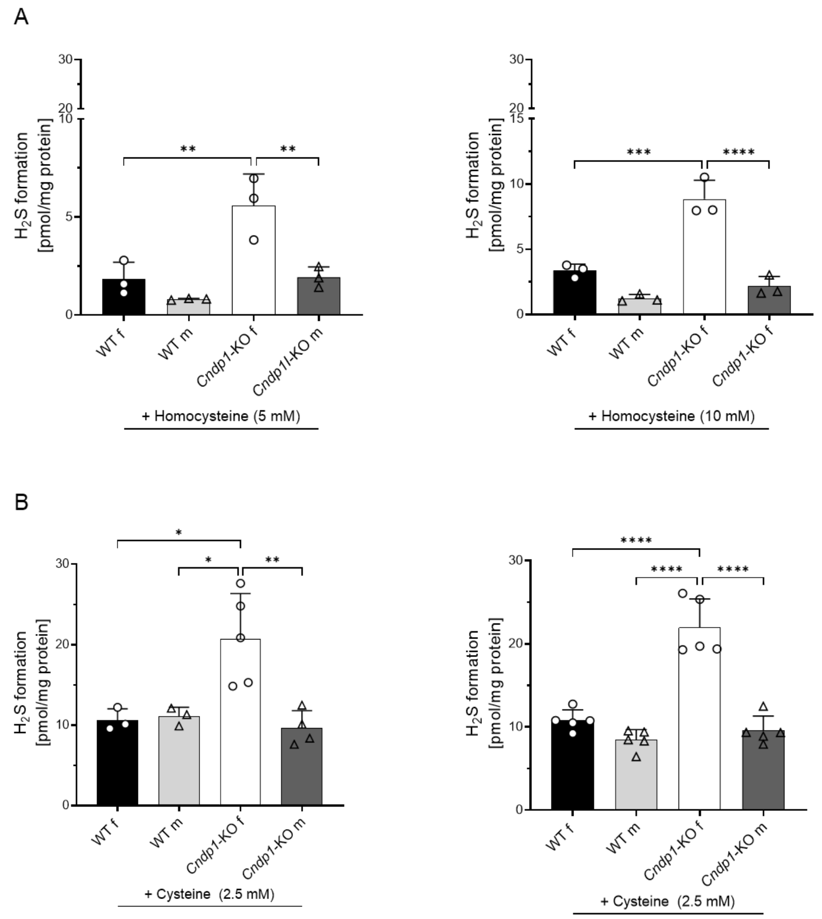

Supplementary Figure S4: Ex vivo renal H_2S formation in *Cndp1*-KO mice

A. Sex-specific kidney H_2S formation capacity in *Cndp1*-WT and -KO mice from homocysteine (A, 47- to 51-week-old), demonstrating similar findings as with addition of cysteine (Figure 4).

B. Ex-vivo H_2S formation from cysteine was similar in kidneys from non-fasting mice (left panel, 23- to 25-week-old) and from mice fasting for 5 hours (right panel, 23- to 25-week-old).

*: p<0.05; **: p<0.01, ***: p<0.001, ****: p<0.0001 (one-way ANOVA with Tukey's test).

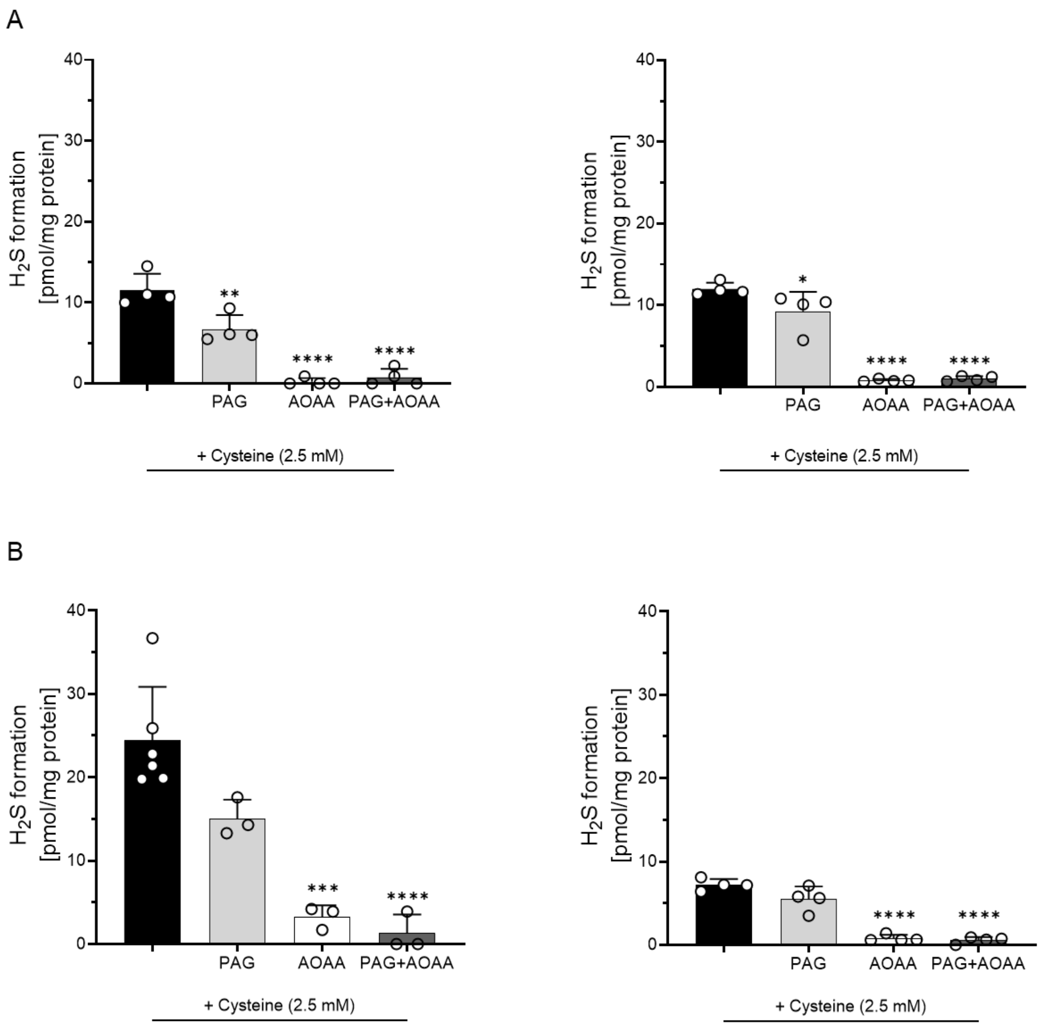

Supplementary Figure S5: Cystathionine-γ-lyase (CSE) and cystathionine-β-synthase (CBS) activity dependent kidney H_2S formation

A. Incubation of mouse kidney tissue lysate with the CSE-inhibitor (0.0315 mM DL-Propargylglycine; PAG) and with the CBS-inhibitor (0.0315 mM O-(Carboxymethyl)-hydroxylamin-hemihydrochlorid; AOAA) decreased renal H_2S formation in female WT (left panel) and male WT(right panel) (47- to 51-week-old).

B. Incubation of kidney tissue lysate with the CSE-inhibitor and with the CBS-inhibitor decreased renal H_2S formation in female *Cndp1*-KO (left panel) and male *Cndp1*-KO mice (right panel) (47- to 51-week-old). Combined treatment of AOAA with PAG had no additive effect on H_2S formation compared to only AOAA (A+B).

*: $p<0.05$; **: $p<0.01$; ***: $p<0.001$; ****: $p<0.0001$ (one-way ANOVA with Tukey's test).

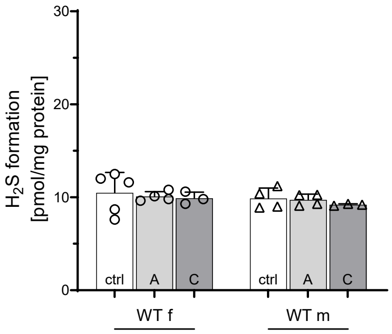

Supplementary Figure S6: Exogenous anserine and carnosine did not affect H_2S formation
Addition of 2.5 mM anserine (A) or carnosine (C) to murine kidney tissue lysate for 6 hours did not increase H_2S formation in female and male WT mice.
